# Mobilization of the *bla*_KPC-14_ gene among heterogenous plasmids in extensively drug-resistant hypervirulent *Klebsiella pneumoniae*

**DOI:** 10.3389/fmicb.2023.1261261

**Published:** 2023-11-15

**Authors:** Lin Wang, Weiyi Shen, Jiachang Cai

**Affiliations:** Clinical Microbiology Laboratory, The Second Affiliated Hospital of Zhejiang University School of Medicine, Zhejiang University, Hangzhou, China

**Keywords:** *bla*_KPC-14_ gene, ceftazidime/avibactam, CR-hvKp, *Klebsiella pneumoniae*, gene transfer

## Abstract

**Introduction:**

Ceftazidime/avibactam (CZA) is an effective alternative for the treatment of infections caused by KPC-producing carbapenem-resistant *Klebsiella pneumoniae* (CRKP). However, KPC variants with CZA resistance have been observed in clinical isolates, further limiting the treatment options of clinical use.

**Methods:**

In this study, we isolated three KPC-14-producing CRKP from two patients in intensive care units without CZA therapy. The antimicrobial susceptibility was determined using the broth microdilution method. Three CRKP were subjected to whole-genome sequencing to analyze the phylogenetic relatedness and the carriage of antimicrobial resistance genes and virulence factors. Long-read sequencing was also performed to obtain the complete sequences of the plasmids. The horizontal transfer of the *bla*_KPC-14_ gene was evaluated by conjugation experiments.

**Results:**

Three CRKP displayed resistance or reduced susceptibility to ceftazidime/avibactam, colistin, and tigecycline. Single-nucleotide polymorphism (SNP) analysis demonstrated the close phylogenetic distance between these strains. A highly similar IncFII/IncR plasmid encoding *bla*_KPC-14_ was shared by three CRKP, with *bla*_KPC-14_ located in an NTE_KPC_-Ib element with the core region of IS*Kpn27*- *bla*_KPC-14_-IS*Kpn6*. This structure containing *bla*_KPC-14_ was also observed in another *tet*(A)-carrying plasmid that belonged to an unknown Inc-type in two out of three isolates. The horizontal transferability of these integrated plasmids to Escherichia coli EC600 was confirmed by the cotransmission of *tet*(A) and *bla*_KPC-14_ genes, but the single transfer of *bla*_KPC-14_ on the IncFII/IncR plasmid failed. Three CRKP expressed yersiniabactin and carried a hypervirulence plasmid encoding *rmpA2* and aerobactin-related genes, and were thus classified as carbapenem-resistant hypervirulent *K. pneumoniae* (hvKP).

**Discussion:**

In this study, we reported the evolution of a mosaic plasmid encoding the *bla*_KPC-14_ gene via mobile elements in extensively drug-resistant hvKP. The *bla*_KPC-14_ gene is prone to integrate into other conjugative plasmids via the NTE_KPC_-Ib element, further facilitating the spread of ceftazidime/avibactam resistance.

## 1. Introduction

The widespread of carbapenem-resistant *Klebsiella pneumoniae* (CRKP) is considered an urgent threat to public health, as it complicates patient care and increases morbidity and mortality in cases of infection (Wang M. et al., 2022; Pérez-Galera et al., 2023). Available data from the China Antimicrobial Surveillance Network (CHINET^1^) showed that the prevalence of CRKP has rapidly increased in China, from 2.9% in 2005 to 24.2% in 2022. Colistin and tigecycline constitute some of the last resorts for the treatment of CRKP infections, however, resistance to these antibiotics in CRKP strains has also been reported recently, further reducing the repertoire of useful antibiotics (Chen et al., 2021; Tian et al., 2021).

Ceftazidime/avibactam (CZA), a novel β-lactam/β-lactamase inhibitor combination, is an effective alternative for the treatment of CRKP infections (Van Duin and Bonomo, 2016). This combination shields ceftazidime from breakdown by Ambler class A, class C, and some class D β-lactamases and thus exhibits potent inhibition of strains producing KPC and OXA-48-like carbapenemases (Criscuolo and Trecarichi, 2020). KPC-producing CRKP is widespread globally and is the predominant type of CRKP in China, which is frequently related to nosocomial outbreaks (Findlay et al., 2021; Wang L. et al., 2022; Wang M. et al., 2022). Although recent studies have shown evidence for CZA as a promising option for such infections, resistance to this antibiotic has rapidly evolved, mainly due to the production of variants of KPC-2 or KPC-3 enzymes (Humphries and Hemarajata, 2017; El-Kady et al., 2022). The single amino acid substitution that confers CZA resistance was commonly encountered in the omega loop (positions 164–179), particularly for the Asp179Tyr (D179Y) mutation in KPC-3 (KPC-31) and KPC-2 (KPC-33) (Livermore et al., 2015; Barnes et al., 2017). Additionally, KPC variants with CZA-resistance mediated by amino acid changes outside the omega loop region (e.g., KPC-41, KPC-23, KPC-14, KPC-8, KPC-123, and KPC-93) were also observed in the clinical isolates from patients following CZA therapy and those who were not treated with CZA (Bianco et al., 2021; Liu et al., 2022b; Wang L. et al., 2022). KPC-14, the variant with a deletion of two amino acids (Δ242-GT-243) of KPC-2 that exhibits CZA resistance, has been sporadically detected in clinical isolates of CRKP (Bianco et al., 2020; Niu et al., 2020; Linh et al., 2021; Jiang et al., 2022).

Here, we investigated the genetic relationship of CRKP harboring two structurally distinct *bla*_KPC-14_-encoding plasmids and analyzed the evolution of one plasmid that was able to undergo horizontal transfer between *Enterobacterales*.

## 2. Materials and methods

### 2.1. Patients and bacterial strains

Three CRKP (strains SP1023 and F1025 from Patient A, and strain SP1030 from Patient B) were isolated from two patients admitted to the neurology intensive care unit (NICU) of a tertiary hospital in Hangzhou City in 2022. Species identification was determined by MALDI-TOF MS (Bruker Daltonik GmbH, Bremen, Germany). This study was approved by the Ethics Committee of The Second Affiliated Hospital of Zhejiang University School of Medicine.

**Figure 1:**
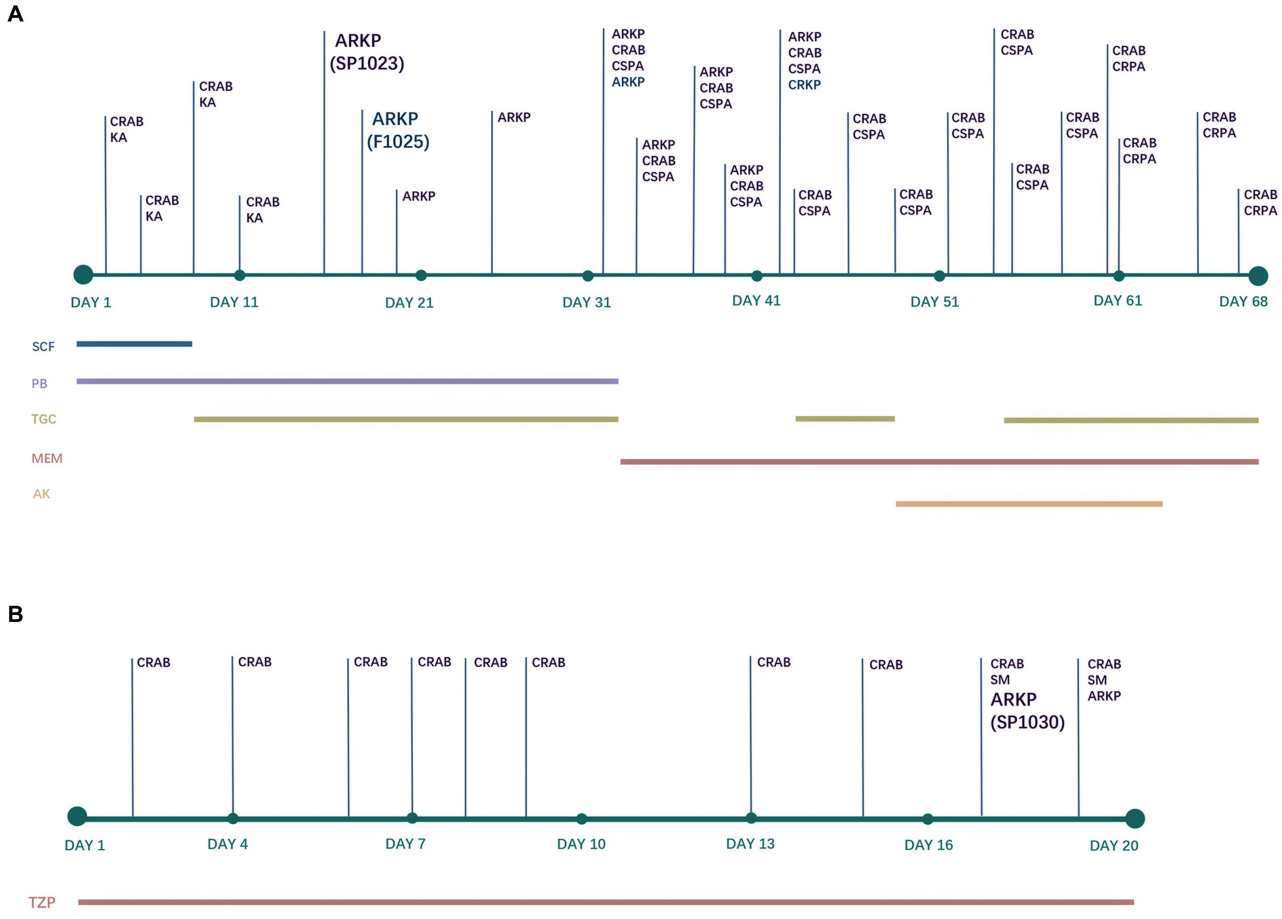
Antimicrobial treatments and bacterial isolation of Patient A **(A)** and Patient B **(B)**. CRAB, carbapenem-resistant *Acinetobacter baumannii*; KA, *Klebsiella aerogenes*; ARKP, ceftazidime/avibactam-resistant *Klebsiella pneumoniae*; CSPA, carbapenem-susceptible *Pseudomonas aeruginosa*; CRPA, carbapenem-resistant *Pseudomonas aeruginosa*; CRKP, carbapenem-resistant *Klebsiella pneumoniae* (ceftazidime/avibactam-susceptible); SM, *Stenotrophomonas maltophilia*; SCF, cefoperazone/sulbactam; PB, polymyxin B; TGC, tigecycline; MEM, meropenem; AK, amikacin; TZP, piperacillin/tazobactam.

Patient A, a 38-year-old male, had poorly controlled hypertension for several years. He underwent three consecutive intracranial hematoma evacuations at a local hospital due to cerebellar hemorrhage. Blood cultures revealed carbapenem-resistant *Acinetobacter baumannii* (CRAB) and a combination of cefoperazone/sulbactam (1,1, 2 g IV every 6 h) and polymyxin B (750,000 IU IV every 12 h) was administered (Figure 1A). The patient was in a coma and was transferred to the NICU of our hospital for further treatment. Upon admission, the patient received supportive treatments, including mechanical ventilation, blood transfusion, fluid replacement, and appropriate medications. The original antimicrobial therapy regimen was continued. The subsequent sputum culture revealed the growth of *Klebsiella aerogenes* and cefoperazone/sulbactam- and carbapenem-resistant *A. baumannii*. Therefore, cefoperazone/sulbactam was replaced with tigecycline (100 mg IV every 12 h) on Day 7. Nine days later (Day 16), both organisms were cleared. However, CRKP (strain SP1023) exhibiting ceftazidime/avibactam and polymyxin B resistance was isolated from the sputum sample (Figure 1A). On Day 18, fecal screening for CRE yielded pandrug-resistant *K. pneumoniae* (strain F1025), which was resistant to ceftazidime/avibactam, polymyxin B, and tigecycline (Figure 1A). In addition to CRKP, *Pseudomonas aeruginosa* and carbapenem-resistant *A. baumannii* (CRAB) were detected in the sputum sample on Day 32. Meropenem was used (1 g IV every 12 h) instead of the previous antimicrobials, and only CRKP was cleared after 9 days of treatment. Tigecycline was used again, and amikacin (400 mg nasogastric feeding every 12 h) was added to the treatment regimen 5 days later. However, *P. aeruginosa* that developed carbapenem resistance and CRAB persisted in the patient’s respiratory tract. Due to his extremely poor condition and acute exacerbation of chronic renal failure, the patient died of multiple organ failure on Day 68.

Patient B, a 40-year-old male with spontaneous intracerebral hemorrhage, underwent surgical evacuation of the intracranial hematoma at a local hospital. Six days later, he remained in a coma and was transferred to the NICU of our hospital. The CT scans showed postoperative hematoma in the surgical area and scattered infiltrates in both lungs. The patient received supportive treatments to reduce intracranial pressure, sedation, pain management, enteral nutrition, endotracheal intubation, and mechanical ventilation. Additionally, piperacillin/tazobactam (8:1, 4.5 g IV every 6 h) was administered for antimicrobial therapy throughout the hospitalization period (Figure 1B). On the second day of admission, CRAB was isolated from the sputum sample and persisted until discharge. On Day 17 and Day 19, CRKP (strain SP1030) was detected in the sputum culture that was resistant to ceftazidime/avibactam and colistin (Figure 1B). After 8 days of mechanical ventilation, the patient was weaned off the ventilator (Day 15). Since the patient’s vital signs stabilized and his mental function recovered, he was discharged for further treatment at a rehabilitation hospital on Day 20.

### 2.2. Antimicrobial susceptibility tests

The minimal inhibitory concentrations (MICs) of 18 antimicrobial agents, including imipenem, meropenem, ertapenem, ceftazidime/avibactam, ceftazidime, cefotaxime, cefepime, piperacillin/tazobactam, cefoperazone/sulbactam, cefmetazole, aztreonam, ciprofloxacin, amikacin, chloramphenicol, fosfomycin, tetracycline, tigecycline, and colistin, were determined using the broth microdilution method (Clinical and Laboratory Standards Institute, 2018) and interpreted according to the Clinical and Laboratory Standards Institute (CLSI) guidelines (Clinical and Laboratory Standards Institute, 2021). Tigecycline susceptibility was interpreted using breakpoints recommended by the US Food and Drug Administration.^2^ *Escherichia coli* ATCC 25922, *K. pneumoniae* 700603, and *Pseudomonas aeruginosa* ATCC 27583 were used as the quality control strains in parallel.

### 2.3. Whole genome sequencing and genome analysis

To investigate the evolution and genetic relatedness of these CRKP, the genomic DNA of three CRKP (strains SP1023, F1025, and SP1030) was subjected to WGS by both the short-read Illumina NovaSeq 6000 platform and the hybrid long-read Oxford Nanopore PromethION 48 platform. The complete genome was assembled by Flye assembler v2.9.2 (Kolmogorov et al., 2020) and polished by Pilon v1.24 (Walker et al., 2014). The antimicrobial resistance genes and the plasmid types for the assembly scaffolds were identified by ResFinder 4.1 and PlasmidFinder 2.0, respectively, at the Center for Genomic Epidemiology.^3^ The sequence types and virulence factors were identified using Kleborate v0.3.0 (Lam et al., 2021). A pairwise comparison of genomes and variant callings for single-nucleotide polymorphisms (SNPs) was conducted using Snippy v4.4.5 with default settings. The plasmids encoding *bla*_KPC-14_ and virulence-associated genes were annotated by the RAST server (Overbeek et al., 2014) and BLASTN program. The comparison of plasmids was visualized and annotated by BRIG v0.95 (Alikhan et al., 2011).

### 2.4. Conjugation experiment

The transferability of *bla*_KPC-14_ genes was estimated by conjugation experiments with filter mating methods (Cai et al., 2008). Rifampin-resistant *E. coli* EC600 was used as the recipient strain. The putative transconjugants grown on selective media supplemented with 8 mg/L CZA or 30 mg/L tetracycline were identified by MALDI-TOF MS and screened for the presence of *bla*_KPC-14_ genes. The conjugation frequency equaled the number of transconjugants divided by the number of recipients.

### 2.5. Virulence testing in the *Galleria mellonella* infection model

The *G. mellonella* (wax moth larvae) infection model was used to confirm the hypervirulent phenotype of the CRKP strains as previously described (McLaughlin et al., 2014). Overnight cultures of *K. pneumoniae* were diluted in sterile phosphate-buffered saline to obtain a concentration of 10^8^ CFU/mL. Wax moth larvae weighing 250–300 mg (Tianjin Huiyude Biotech Company, Tianjin, China) were injected with 10 μL bacterial suspension and incubated for 48 h at 35°C. The survival rate of *G. mellonella* was recorded at 12 h, 24 h, 36 h, and 48 h. ST11 *K. pneumoniae* FJ8 without virulence factors and the hypervirulent *K. pneumoniae* 4 were used as the negative and positive controls, respectively (Gu et al., 2018). All experiments were performed in triplicate. Kaplan–Meier survival curves were plotted using Prism 9.

### 2.6. Nucleotide sequence accession numbers

The complete genome of the chromosome and plasmids for *K. pneumoniae* SP1023, F1025, and SP1030 were downloaded with BioSample accession numbers SAMN36464959, SAMN36465167, and SAMN36465169, respectively.

## 3. Results

### 3.1. Antimicrobial susceptibility results

As Table 1 illustrated, three CRKP shared a high-level resistance to CZA with MIC values of >64/4 mg/L and showed resistance or decreased susceptibility to meropenem and ertapenem. These strains also shared an extensive drug resistance (XDR) profile (Magiorakos et al., 2012) to ceftazidime, cefotaxime, cefepime, aztreonam, ciprofloxacin, amikacin, chloramphenicol, fosfomycin, and tetracycline but retained susceptibility to imipenem. More worrisome, resistance to colistin was observed in these CRKP, and *K. pneumoniae* F1024 exhibited additional resistance to another clinically important antibiotic, tigecycline, which was interpreted as resistance to almost all the antimicrobial agents frequently used in clinical settings.

**Table 1:** Antimicrobial susceptibility results of *K. pneumoniae* isolates and their *E. coli* transconjugants.

Strain	MICs (mg/L)																	
	IPM^b^	MEM	ETP	CZA	CAZ	CTX	FEP	TZP	SCF	CMZ	ATM	CIP	AK	CHL	FOS	TE	TGC	COL
*K. pneumoniae* SP1023	0.25	2	4	>64/4	>128	64	>64	32/4	32/16	32	>128	>32	>128	>128	>256	>64	2	4
*K. pneumoniae* F1025	0.5	4	16	>64/4	>128	>128	>64	>256/4	64/32	128	>128	>32	>128	>128	>256	>64	8	4
*K. pneumoniae* SP1030	0.5	2	8	>64/4	>128	>128	>64	>256/4	128/64	32	>128	>32	>128	>128	>256	>64	2	4
Transconjugant SP1023-TE^a^	0.25	0.06	≤0.03	≤0.5/4	≤0.5	≤0.5	≤0.5	≤8/4	≤8/4	≤2	≤1	1	≤4	>64	≤8	>64	0.125	≤0.5
Transconjugant F1025-CZA	0.25	0.06	0.25	16/4	>128	32	16	≤8/4	≤8/4	≤2	>128	1	≤4	>64	≤8	>64	0.125	≤0.5
Transconjugant F1025-TE	0.25	0.06	0.25	16/4	>128	32	16	≤8/4	≤8/4	≤2	>128	1	≤4	>64	≤8	>64	0.125	≤0.5
Transconjugant SP1030-CZA	0.25	0.06	0.5	16/4	>128	32	32	≤8/4	≤8/4	≤2	>128	1	≤4	>64	≤8	>64	0.125	≤0.5
Transconjugant SP1030-TE	0.25	0.06	0.5	16/4	>128	32	32	≤8/4	≤8/4	≤2	>128	1	≤4	>64	≤8	>64	0.125	≤0.5
*E. coli* EC600	0.25	0.06	≤0.03	≤0.5/4	≤0.5	≤0.5	≤0.5	≤8/4	≤8/4	≤2	≤1	≤0.25	≤4	≤4	≤8	≤1	0.06	1

a *E. coli* transconjugants were selected by two different agar plates containing ceftazidime/avibactam or tetracycline, respectively.

b IPM, imipenem; MEM, meropenem; ETP, ertapenem; CZA, ceftazidime/avibactam; CAZ, ceftazidime; CTX, cefotaxime; FEP, cefepime; TZP, piperacillin/tazobactam; SCF, cefoperazone/sulbactam; CMZ, cefmetazole; ATM, aztreonam; CIP, ciprofloxacin; AK, amikacin; CHL, chloramphenicol; FOS, fosfomycin; TE, tetracycline; TGC, tigecycline; COL, colistin.

For piperacillin/tazobactam and ceftazidime/avibactam, the tazobactam and avibactam were tested at a fixed concentration of 4 mg/L. For cefoperazone/sulbactam, the combination was tested with concentrations of 2:1 ratio (antibiotic: inhibitor).

### 3.2. Whole genome analysis of KPC-14-producing isolates

All three CRKP were identified as the ST11 type, which was the predominant ST type of CRKP in China (Liu et al., 2022a). Moreover, these three strains all belonged to the K64 serotype. Genome-based phylogenetic analysis suggested that these CRKP were closely related within 27 SNPs (with strain F1025 as the reference genome), indicating that these XDR strains originated from the same clone.

Whole-genome analysis demonstrated that three CRKP exhibited a similar carriage profile of β-lactamase genes, including *bla*_KPC-14_, *bla*_SHV-11_, *bla*_TEM-1_, and *bla*_LAP-2_, while *K. pneumoniae* F1025 and SP1030 additionally expressed SHV-12 (Table 2). However, the amplification of the *bla*_KPC_ gene failed both in *A.baumannii* and *P. aeruginosa* isolated from Patient A. Multiple antimicrobial resistance genes were also identified in three CRKP, conferring resistance to quinolones (*qnrS1*), phenicols (*catA2*), sulfonamides (*sul1* and *sul2*), tetracyclines [*tet*(A)], fosfomycin (*fosA* and *fosA3*), and aminoglycosides (*aadA2b* and *rmtB*). Detailed analysis showed three CRKP shared that mutations for type 1 Tet(A) variants (I5R, V55M, I75V, T84A, S201A, F202S, and V203F) and the insertion of IS*Kpn26* elements at position 75 in the *acrR* gene, both of which contributed to the elevated MICs of tigecycline (Chiu et al., 2017). Insertional inactivation by IS*Kpn18* was also detected in *ramR*, another tigecycline resistance determinant gene, further driving the generation of resistance to this antibiotic in *K. pneumoniae* SP1025. The *mgrB* genes was interrupted by IS*Kpn26* at the same position (nucleotide 75) in three CRKP, thus accounting for the resistance to colistin.

**Table 2:** Clinical and genetic characteristics of three KPC-14-producing *K. pneumoniae* isolates.

Strain	Patient	Gender	Age	Diagnosis	Specimen	Antibiotic resistance genes	Virulence factors	*mgrB* mutation	*ramR* mutation	*acrR* mutation
*K. pneumoniae* SP1023	Patient A	Male	38	Cerebral hemorrhage	Sputum	*bla*_KPC-14_, *bla*_SHV-11_, *bla*_TEM-1_, *bla*_LAP-2_, *qnrS1*, *catA2*, *tet*(A), *fosA*, *fosA3*, *sul1*, *sul2*, *aadA2b*, *rmtB*	RmpA2, aerobactin, yersiniabactin	IS*Kpn26* insertion at nt 75	Wild type	IS*Kpn26* insertion at nt 281
*K. pneumoniae* F1025	Patient A	Male	38	Cerebral hemorrhage	Feces	*bla*_KPC-14_, *bla*_SHV-11_, *bla*_SHV-12_, *bla*_TEM-1_, *bla*_LAP-2_, *qnrS1*, *catA2*, *tet*(A), *fosA*, *fosA3*, *sul1*, *sul2*, *aadA2b*, *rmtB*	RmpA2, aerobactin, yersiniabactin	IS*Kpn26* insertion at nt 75	IS*Kpn18* insertion at nt 396	IS*Kpn26* insertion at nt 281
*K. pneumoniae* SP1030	Patient B	Male	40	Cerebral hemorrhage	Sputum	*bla*_KPC-14_, *bla*_SHV-11_, *bla*_SHV-12_, *bla*_TEM-1_, *bla*_LAP-2_, *qnrS1*, *catA2*, *tet*(A), *fosA*, *fosA3*, *sul1*, *sul2*, *aadA2b*, *rmtB*	RmpA2, aerobactin, yersiniabactin	IS*Kpn26* insertion at nt 75	Wild type	IS*Kpn26* insertion at nt 281

### 3.3. Transferability of *bla*^KPC-14^-carrying plasmids

The *bla*_KPC-14_ gene could be conjugated into *E. coli* EC600 from *K. pneumoniae* F1025 and SP1030 with similar conjugation efficiencies of 2.1 × 10^−5^ and 3.5 × 10^−5^, respectively, but conjugation failed in strain SP1023, suggesting a different location of the *bla*_KPC-14_ gene among these strains. Elevated CZA and several β-lactam MIC values were observed in *E. coli* transconjugants for *K. pneumoniae* F1025 and SP1030, confirming the functionality of *bla*_KPC-14_ (Table 1). Notably, these *E. coli* transconjugants also showed resistance to tetracycline and slightly higher tigecycline MIC values, indicating the possible cotransfer of *tet*(A) and *bla*_KPC-14_ genes. Therefore, selective media containing tetracycline were used to screen for putative transconjugants. Each of the three CRKP was able to transfer the *tet*(A) gene to the recipient *E. coli* EC600 with a similar efficiency at approximately 10^−5^; however, *E. coli* transconjugants with different donors displayed heterogeneity in antimicrobial susceptibility profiles. Unlike the *E. coli* transconjugants F1025-TE and SP1030-TE (with *K. pneumoniae* F1025 and SP1030 as donors, respectively), which displayed similar profiles to those of their counterparts for *bla*_KPC-14_, the *E. coli* transconjugant SP1023-TE (with *K. pneumoniae* SP1023 as the donor) showed decreased susceptibility to tetracycline and tigecycline but retained the same MIC values for CZA and other β-lactams as the recipient strain. The amplification of *bla*_KPC-14_ was also carried out in *E. coli* transconjugants F1025-TE and SP1030-TE, but failed in SP1023-TE, further supporting the dissimilar *bla*_KPC-14_ gene locations and plasmid carriage of the three CRKP.

### 3.4. Molecular analysis of *bla*_KPC-14_-carrying plasmids

**Figure 2:**
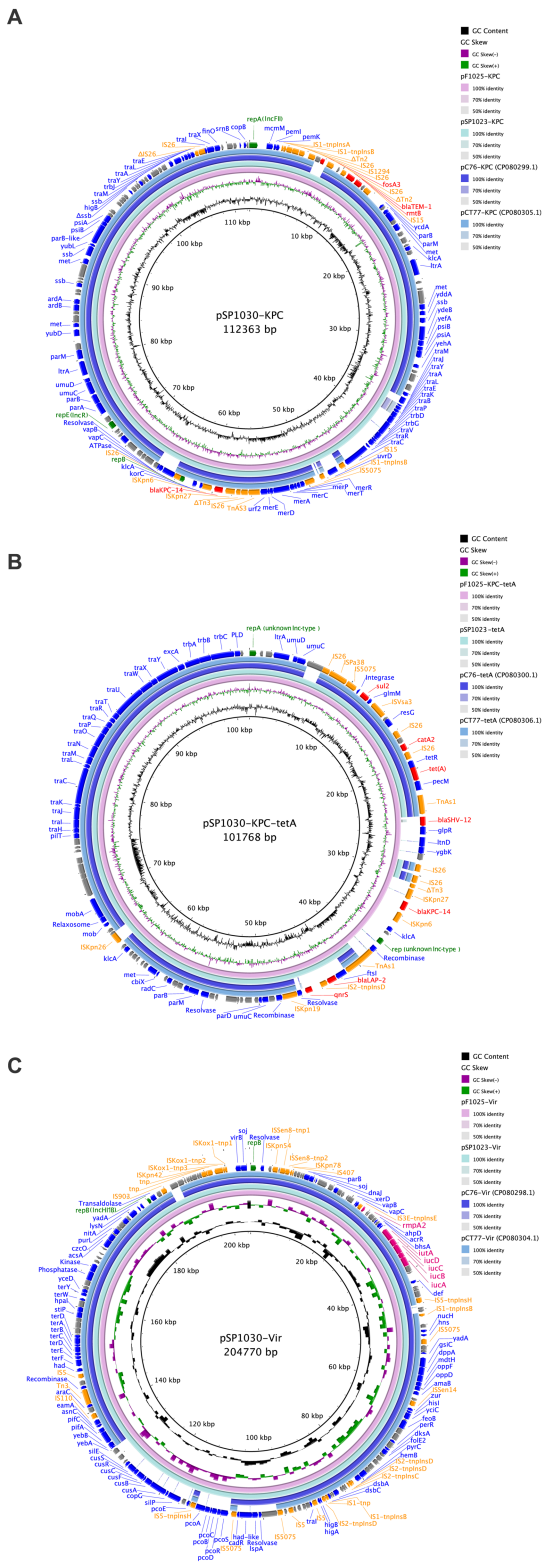
Comparisons of *bla*_KPC_-carrying plasmid **(A)**, *tet*(A)-carrying plasmid **(B)**, and the virulence plasmid **(C)** with the reference plasmids. The circles from inside to outside display the scale in kilobase pairs, the GC skew, the GC content, the similarity to the reference plasmids, and the annotation of the plasmid in our study, respectively. All insertion sequences are labeled orange, antimicrobial resistance genes are labeled red, genes encoding proteins with known functions are labeled blue, *rep* genes are labeled green, and the hypothetical proteins are labeled gray.

To clarify the location and genetic platforms of *bla*_KPC-14_ genes, hybrid long-read sequencing of all three strains was performed. This yielded the complete genome for three CRKP with similar sizes of approximately 6 Mbp, consisting of one chromosome and varied numbers of plasmids (Table 3). A similar IncFII/IncR hybrid plasmid encoding *bla*_KPC-14_ was harbored by all three CRKP, designated as plasmids pSP1023-KPC, pF1025-KPC, and pSP1030-KPC for *K. pneumoniae* SP1023, F1025, and SP1030, respectively. These plasmids carried a variety of additional antimicrobial resistance determinants, including the *bla*_TEM-1_, *fosA3*, and *rmtB* genes, with sizes ranging from 112,363–112,364 bp. Blast analysis demonstrated that the backbone of this plasmid showed the highest similarity to plasmid pCRKP66R-3 with 100% identity (100% coverage, GenBank accession CP063835), which harbored the *bla*_KPC-2_ gene. These plasmids also displayed 100% identity to two other *bla*_KPC-2_-encoding plasmids named plasmid pC76-KPC (86% coverage, GenBank accession CP080299) and pCT77-KPC (87% coverage, GenBank accession CP080305), both of which were previously discovered in CRKP in our hospital (Figure 2) (Chen et al., 2021). The *bla*_KPC-14_ gene was in the non-Tn*4401* element as an NTE_KPC_-Ib-like transposon with a genetic array of “IS*26*-ΔTn*3*-IS*Kpn8*-*bla*_KPC-14_-IS*Kpn6*-*korC-klcA-rep-*orf-IS*26*,” which was commonly and uniquely identified in ST11 KPC-producing CRKP (Yang et al., 2021). Consistent with many IncFIIK/IncR *bla*_KPC_-harboring plasmids widely disseminated in China, this plasmid was nonconjugative, which was probably due to the absence of relaxase, thus explaining the failure of the single transfer of *bla*_KPC-14_ in the conjugation experiment.

**Table 3:** Location of antimicrobial resistance genes and virulence genes.

Strain	Patient	Chromosome	*bla*_KPC_-carrying plasmid	*tet*(A)-carrying plasmid	Virulence plasmid
*K. pneumoniae* SP1023	Patient A	4,747,758 bp, *bla*_SHV-11_, *fosA*, *sul1*, *aadA2b*, *irp1*, *irp2* No. of SNPs: 3^a^	112,364 bp, nonconjugative, *bla*_KPC-14_, *bla*_TEM-1_, *fosA3*, *rmtB* No. of SNPs: 0	84,754 bp, conjugative, *tet*(A), *catA2*, *bla*_LAP-2_, *qnrS1*, *sul2* No. of SNPs: 1	204,778 bp, *rmpA2*, *iutA*, *iucABCD* No. of SNPs: 0
*K. pneumoniae* F1025	Patient A	5,424,169 bp, *bla*_SHV-11_, *fosA*, *sul1*, *aadA2b*, *irp1*, *irp2* No. of SNPs: NA	112,364 bp, nonconjugative, *bla*_KPC-14_, *bla*_TEM-1_, *fosA3*, *rmtB* No. of SNPs: NA	101,767 bp, conjugative, *tet*(A), *catA2*, *bla*_LAP-2_, *qnrS1*, *sul2*, *bla*_KPC-14_, *bla*_SHV-12_ No. of SNPs: NA	204,770 bp, *rmpA2*, *iutA*, *iucABCD* No. of SNPs: NA
*K. pneumoniae* SP1030	Patient B	5,477,323 bp, *bla*_SHV-11_, *fosA*, *sul1*, *aadA2b*, *irp1*, *irp2* No. of SNPs: 4	112,363 bp, nonconjugative, *bla*_KPC-14_, *bla*_TEM-1_, *fosA3*, *rmtB* No. of SNPs: 1	101,768 bp, conjugative, *tet*(A), *catA2*, *bla*_LAP-2_, *qnrS1*, *sul2*, *bla*_KPC-14_, *bla*_SHV-12_ No. of SNPs: 1	204,770 bp, *rmpA2*, *iutA*, *iucABCD* No. of SNPs: 0

a The SNP numbers was estimated by Snippy with the reference sequences of *K. pneumoniae* F1025.

No., numbers; NA, not applicable.

The type 1 Tet(A) variant colocalized with the *catA2*, *bla*_LAP-2_, *qnrS1*, and *sul2* genes on unknown Inc-type plasmids with sizes of 84,754 bp, 101,767 bp, and 101,768 bp for *K. pneumoniae* SP1023, F1025, and SP1030, respectively (Figure 2A). This backbone was also observed in *tet*(A)-harboring plasmids in *K. pneumoniae* C76 and *K. pneumoniae* CT77 with 100% identity (91% coverage) but integrated with a 4,956 bp DNA fragment (based on *K. pneumoniae* SP1030) containing the *bla*_LAP-2_ and *qnrS1* genes. Unlike plasmid pSP1023-tetA in *K. pneumoniae* SP1023, the *bla*_KPC-14_ locus of 8,248 bp was inserted into the *tet*(A)-carrying plasmids in *K. pneumoniae* F1025 and SP1030, which were further designated as plasmid pF1025-KPC-tetA and pSP1030-KPC-tetA, respectively. Another resistance locus harboring the *bla*_SHV-12_ gene was also observed in these two plasmids but was absent in plasmid pSP1023-tetA. The structural discrepancy in these *tet*(A)-carrying plasmids echoed the differences in the genetic and phenotypic profiles of the transconjugants we noticed. These observations indicated that the plasmids carrying both *tet*(A) and *bla*_KPC-14_ genes possibly evolved from those with a single occurrence of the *tet*(A) gene, such as pSP1023-tetA, which could also be further traced back to previously identified plasmids. The *bla*_KPC-14_ genes were flanked by an NTE_KPC_-Ib-like transposon similar to that on the IncFII/IncR plasmids in our study, indicating that these nonconjugative plasmids might be the source of the *bla*_KPC-14_-carrying fragments for plasmids pF1025-KPC-tetA and pSP1030-KPC-tetA. As previously reported, the diversity of mobile elements and transposons in NTE_KPC_-I elements actively promoted the transposition of the *bla*_KPC_ genes to various genetic locations (Yang et al., 2021); thus, we speculated that the mobilization event mediated the integration of the *bla*_KPC-14_ locus into the backbones of *tet*(A)-carrying plasmids, thus creating the binary carriage profile of *bla*_KPC-14_-harboring plasmids in *K. pneumoniae* F1025 and SP1030. The integrated plasmids retained the fully functional conjugative genes homologous to the previous *tet*(A)-carrying plasmids (Figure 2), further facilitating the cotransfer and dissemination of KPC-14 and the type 1 Tet(A) variant among *Enterobacterales*.

### 3.5. Virulence analysis

**Figure 3:**
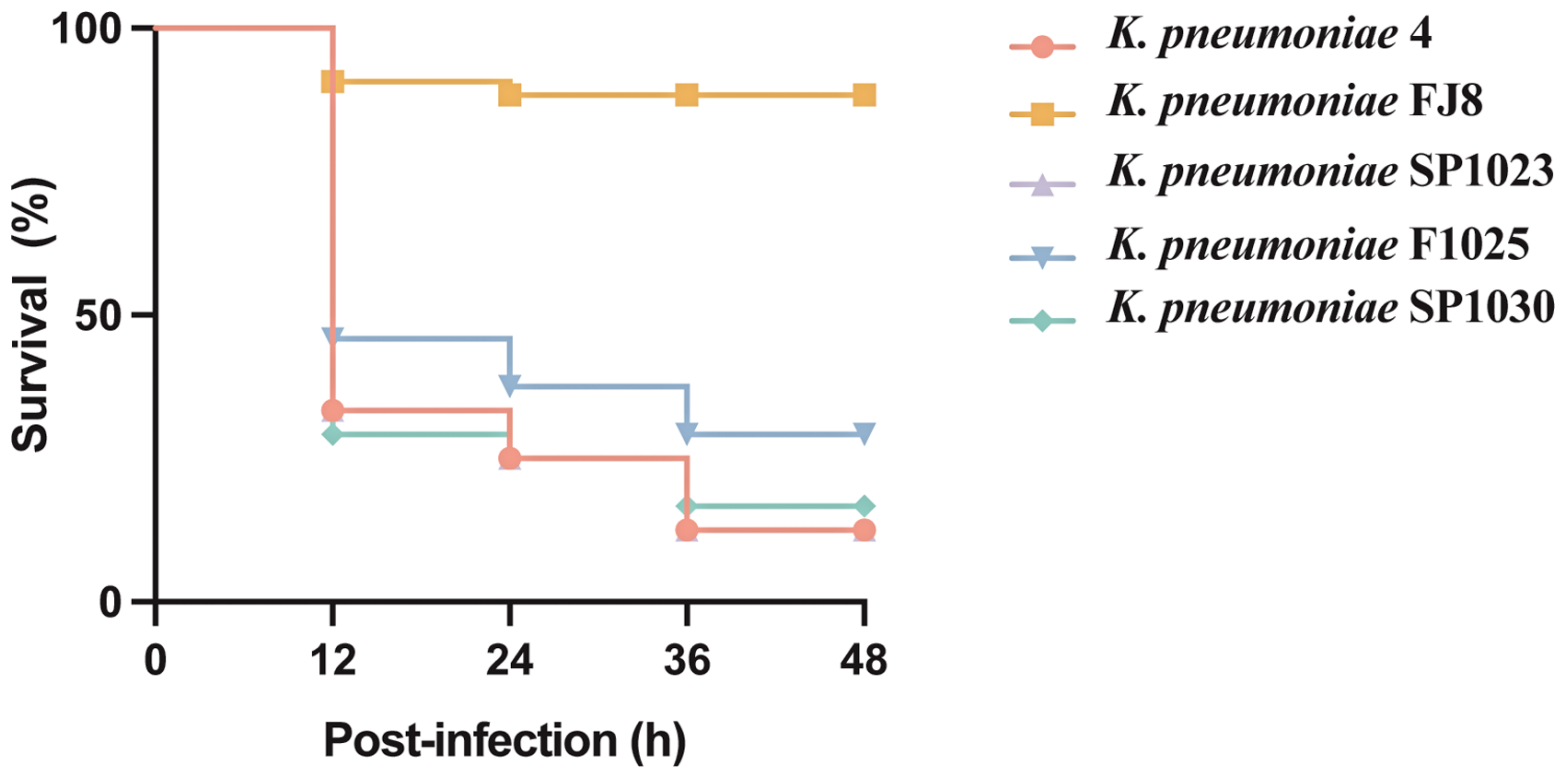
Virulence potential of *K. pneumoniae* strains in a *G. mellonella* infection model.

The hypervirulent phenotype of three CRKP was observed in the *G. mellonella* infection model (Figure 3). At 48 h post-infection, the survival rates of larvae infected by strains SP1023, F1025, and SP1030 were 12.5, 29.2, and 16.7%, respectively, which were lower than that of larvae infected by the negative control strain *K. pneumoniae* FJ8 at 79.2%. WGS analysis showed that these strains expressed the same virulence factor profile specific to hypervirulent *K. pneumoniae* (hvKP), including RmpA2, aerobactin, and yersiniabactin (Table 2); thus, the CRKP in our study were classified as CR-hvKP. Long-read sequencing revealed that the *rmpA2* and the *iutAiucABCD* gene cluster (aerobactin siderophore) were located on the IncHI1B/IncFIB-type pLVPK-like plasmid with sizes ranging from 204,770 to 204,778 bp (Table 3, Figure 2C). These plasmids showed high identity (>99%) to a variety of other hypervirulence plasmids of *K. pneumoniae* in the NCBI database, indicating the wide dissemination of these plasmids conferring hypervirulent phenotypes.

## 4. Discussion

The rapid and wide dissemination of KPC-producing CRKP represents a serious threat to public health and a serious challenge for healthcare workers. Most of the KPC-producing CRKP also harbor determinants that confer resistance to a variety of antimicrobial agents thus further limiting the clinical options for treatment. CZA exhibited great activity against these multidrug-resistant pathogens; however, many concerns have been raised over the emergence of resistant KPC variants with various genetic landscapes, demonstrating the substantial evolutionary potential of this enzyme (Findlay et al., 2021; Liu et al., 2022b; Wu et al., 2022).

In this study, we described the emergence of the CZA-resistant CRKP harbored the *bla*_KPC-14_ gene on two structurally different plasmids. Three CRKP collectively harbored a *bla*_KPC-14_-carrying IncFII/IncR plasmid, which was widespread and commonly identified in KPC-producing CRKP in China (Chen et al., 2014; Dong et al., 2018). There were a few studies that described the emergence and *in vivo* selection of KPC-14 rendering resistance following CZA treatment (Bianco et al., 2020; Niu et al., 2020). However, due to the absence of CZA therapeutic regimens during the hospitalization of the two patients, the source of the *bla*_KPC-14_ gene in our hospital was still unclear. As recently shown, the KPC-14 enzyme demonstrated the loss of carbapenemase activity (Compain and Arthur, 2017), which was true for the low-level resistance or susceptibility to the carbapenems of KPC-14 producers in our study. This finding reminded us that these resistance determinants may silently spread and be easily ignored during routine surveillance in clinical settings. Although these *bla*_KPC-14_-encoding plasmids were nonconjugative, they could provide translocatable fragments as reservoirs for the mobilization of the *bla*_KPC-14_ gene into other plasmid backbones. In this study, we also identified a conjugative plasmid integrated with the *bla*_KPC-14_-containing NTE_KPC_-I element, which was relatively prevalent among clinical strains in China (Cerdeira et al., 2019). Structurally similar plasmids were previously reported in our hospital with the colocation of the type 1 Tet(A) variant (Chen et al., 2021), which was associated with resistance to another clinically important antibiotic, tigecycline. Moreover, the evolution of these plasmids was observed, as they could additionally capture various other resistance loci, further contributing to the coselection and persistence of these determinants of resistance to last-line antibiotics. The considerable genetic plasticity of these plasmids enabled the acquisition of further resistance-encoding and hypervirulence-encoding genetic elements; thus, these isolates can better adapt to various environments to stimulate the spread of *bla*_KPC-14_ among *Enterobacterales*.

The multidimensional transmission and potential silent spread of *bla*_KPC-14_ in CRKP is concerning, especially in those that also carry hypervirulent phenotypes. The KPC-14-producing CRKP in our study originated from the same ST11 clone, which was the dominant clone of CRKP in China and served as a salient example of the evolutionary acquisition of resistance genes and virulence factors for a newly emerged superbug (Liao et al., 2020). The pLVPK-like virulence plasmids commonly converted normal ST11 strains to ST11 hvKP and were first reported in our hospital in 2018 (Gu et al., 2018). These plasmids enhanced the environmental survival and the rapid dissemination of the hypervirulent phenotype with a limited fitness cost in ST11 CRKP (Zhou et al., 2020), consistent with the persistence of these plasmids during spread and evolution observed in our study. More worrisome, these superbugs still have the exceptional ability to attain extra resistance to clinically important drugs such as colistin and tigecycline via *in vivo* selection following antibiotic treatment. This could have affected *K. pneumoniae* F1025 in our study, which displayed resistance to almost all antibiotics, including CZA, colistin, and tigecycline. The emergence of such XDR strains may cause severe infections that are difficult to treat with current antibiotics, especially for ICU patients with complicated diseases.

## 5. Conclusion

In this study, we described the evolution of a conjugative mosaic plasmid encoding the *bla*_KPC-14_ gene via mobile elements in CR-hvKP. The nonconjugative IncFII/IncR plasmid could serve as the reservoir of the mobilizable fragment of the *bla*_KPC-14_ gene, similar to NTE_KPC_-I elements, allowing it to integrate into other conjugative plasmid backbones, further facilitating the spread of *bla*_KPC-14_. Therefore, constant surveillance to control the development and further spread of CZA resistance is of great significance.

## Data availability statement

The datasets presented in this study can be found in online repositories. The names of the repository/repositories and accession number(s) can be found in the article/supplementary material.

## Ethics statement

The studies involving humans were approved by the Ethics Committee of The Second Affiliated Hospital of Zhejiang University School of Medicine. The studies were conducted in accordance with the local legislation and institutional requirements. The human samples used in this study were acquired from primarily isolated as part of your previous study for which ethical approval was obtained. Written informed consent for participation was not required from the participants or the participants’ legal guardians/next of kin in accordance with the national legislation and institutional requirements.

## Author contributions

LW: Data curation, Formal analysis, Investigation, Methodology, Resources, Validation, Writing – original draft. WS: Data curation, Formal analysis, Investigation, Validation, Writing – original draft, Software, Visualization. JC: Conceptualization, Funding acquisition, Project administration, Supervision, Writing – review & editing.

## Footnotes

^1^ http://www.chinets.com/

^2^ https://www.fda.gov/drugs/developmentresources/tigecycline-injection-products

^3^ https://www.genomicepidemiology.org/
